# Ensemble Learning Method for the Continuous Decoding of Hand Joint Angles

**DOI:** 10.3390/s24020660

**Published:** 2024-01-20

**Authors:** Hai Wang, Qing Tao, Xiaodong Zhang

**Affiliations:** 1 School of Mechanical Engineering, Xinjiang University, Urumqi 830017, China; wanghai@stu.xju.edu.cn (H.W.); xdzhang@mail.xjtu.edu.cn (X.Z.); 2 Shaanxi Key Laboratory of Intelligent Robot, Xi’an Jiaotong University, Xi’an 710049, China

**Keywords:** sEMG, hand joint angle, ensemble learning, XGBoost, LightGBM, CatBoost, stacking

## Abstract

Human–machine interface technology is fundamentally constrained by the dexterity of motion decoding. Simultaneous and proportional control can greatly improve the flexibility and dexterity of smart prostheses. In this research, a new model using ensemble learning to solve the angle decoding problem is proposed. Ultimately, seven models for angle decoding from surface electromyography (sEMG) signals are designed. The kinematics of five angles of the metacarpophalangeal (MCP) joints are estimated using the sEMG recorded during functional tasks. The estimation performance was evaluated through the Pearson correlation coefficient (CC). In this research, the comprehensive model, which combines CatBoost and LightGBM, is the best model for this task, whose average CC value and RMSE are 0.897 and 7.09. The mean of the CC and the mean of the RMSE for all the test scenarios of the subjects’ dataset outperform the results of the Gaussian process model, with significant differences. Moreover, the research proposed a whole pipeline that uses ensemble learning to build a high-performance angle decoding system for the hand motion recognition task. Researchers or engineers in this field can quickly find the most suitable ensemble learning model for angle decoding through this process, with fewer parameters and fewer training data requirements than traditional deep learning models. In conclusion, the proposed ensemble learning approach has the potential for simultaneous and proportional control (SPC) of future hand prostheses.

## 1. Introduction

Advancement in sEMG signal intention perception technology has brought about the creation of high-performing sEMG prosthetic hands, offering hope and a better quality of life to those with disabilities caused by amputation. In 1948, Reiter triumphantly constructed the first iteration of prosthesis controlled through sEMG signals. This pioneering feat was a major step forward in the use of electromyography as a means of controlling prosthetic devices. At present, control of sEMG-powered prosthetic hands is primarily based on recognizing a limited number of pre-determined actions. Fang et al. [1] made a noteworthy contribution to the field of sEMG-controlled prosthetics with their proposal of a biologically inspired neural network. Yamanoi et al. [2] tackled a significant challenge in the field of sEMG-controlled prosthetics. They designed a convolutional neural network that allows for the realization of an algorithm model with improved long-term recognition robustness. U. Cote-Allard et al. [3] made a significant impact in the field of sEMG-controlled prosthetics with their innovative use of transfer learning in the training process of machine learning algorithms. Hu et al. [4] made a significant contribution to the field of sEMG-controlled prosthetics through their innovative use of a convolutional loop neural network with an attention mechanism. He et al. [5] used SSA-DBN to design a gait classification model, and the recognition accuracy was improved by about 2% compared with the unoptimized DBN, which was 97.73%. Yao et al. [6] proposed a gait recognition method based on the deep neural network of (sEMG) signals to improve the accuracy of gait recognition, which exceeded 95%. However, one of the major challenges that has been identified is the rapid decline in recognition accuracy as the number of actions increases. To achieve better results in sEMG-controlled prosthetics, it is necessary to develop new methods for decoding the continuous movements of hands.

## 2. Related Work

Many research groups have used machine learning techniques to decode the intricate movements of hands by analyzing sEMG signals to extract motion information. Currently, some researchers have made advancements in decoding continuous angles using sEMG signals. Zhang et al. [7] utilized sparse Gaussian processes to translate sEMG signals from eight forearm muscles into the angles of five-finger joints and achieved an impressive online Pearson correlation coefficient of 0.91. Ma et al. [8] achieved accurate recognition of the continuous angle movements of upper limb joints by employing a stack of long short-term memory neural networks. Deng et al. [9] used a PCA-RELM algorithm to realize angle estimation for knee joint movements, where the correlation coefficients were more than 0.88. Zhu et al. [10] and Dario’s teams [11] obtained reliable motor neuron action potential signals through the use of high-density sEMG signal acquisition equipment, providing a strong foundation for the advanced control of intelligent prosthetic limbs. Ameri et al. [12] were the first to use a convolutional neural network to decode wrist motion sEMG signals in real time, achieving a coefficient determination of 0.99. Lin et al. [13] achieved continuous, real-time control of hand movements by utilizing sparse nonnegative matrix decomposition on the sEMG signals. Ameri et al. [14] utilized a regression convolution neural network for continuous online recognition of upper limb motion. Nielsen et al. [15] and Ning et al. [16] each independently executed experiments involving synchronous proportional control through mirror symmetry training, leading to the successful synchronous proportional control of both the wrist joint torque and angle. Ngeo et al. [17] utilized a combination of musculoskeletal modeling and machine learning techniques, inputting the activation of eight channels of muscle signals into either an artificial neural network or Gaussian processes to successfully decode continuous hand motion. Their methods yielded impressive correlation coefficients of 0.71 and 0.84. Celadon et al. [18] utilized high-density electrodes to decode the mapping relationship between sEMG signals and proportional changes in finger joint angles through the maximum voluntary contraction force (MVC). The team employed the common space mode algorithm and linear discriminant analysis algorithm, achieving an online control with a mean square error of less than 3.6%. Xiloyannis et al. [19] utilized the Gaussian process approach, which leveraged both the previously decoded angle and the current sEMG and mechanomyogram signals, to derive the current finger joint angle. The Pearson correlation coefficient was about 0.6. Bao et al. [20] utilized a hybrid model of convolutional neural network (CNN) and long short-term memory (LSTM) for the purpose of decoding the continuous angle of the wrist. Quivira et al. [21] and Koch et al. [22] successfully employed a recurrent neural network to decode hand movements through regression. Zhang et al. [23] successfully utilized the combination of LSTM and ANN to decode joint angles and forces simultaneously, thus providing an innovative approach for the real-time control of limb movements. Mao et al. [24] proposed a new control scheme, which used SVM, that simultaneously estimates continuous grip force and wrist angles using a combination of sEMG and acceleration (ACC) signals. Most of the above-mentioned research was focusing on deep learning or very simple machine learning methods, and none of them tried ensemble learning methods or stacking learning strategy. The main problem for all of them is that the models are too complex and lack interpretability. Moreover, they are not easy to deploy on embodied systems because of the large computational requirements and storage requirements.

To solve the problem of the low precision of the continuous decoding of finger joint angles based on sEMG signals, our proposed method has tried three mainstream ensemble learning models to decode the angle from sEMG. These three ensemble models are based on the tree model. Referring to the research of [25], we find that the tree model is effective for explaining tabular data. Tree-based models have strong interpretability and robustness in processing tabular data; they can handle missing values and outliers and do not require a lot of data preprocessing and feature engineering, so they can handle tabular data better in some cases. Additionally, we implement the stacking method to combine the three ensemble models to improve the model’s performance. An experimental platform is customized to carry out synchronous acquisition experiments of finger joint angles and sEMG signals. We propose a new machine learning model building scheme for this task, which is efficient and robust. The ensemble learning method as a traditional machine learning method is much faster and cheaper than the deep learning method. For the motion control of the prosthetic hand, decoding the model’s efficiency is very important for the feeling experienced by the amputee. The ensemble method is our choice. In this paper, we give a complete model training scheme to help engineers to find the best decoding method. More importantly, the method also has very low computational requirements and a simple structure, which are very ideal for embodied system like prosthetic hands. For performance judgement, we compare the seven ensemble models that we propose and the traditional machine learning model of the Gaussian process, which is widely used for this kind of task.

## 3. Materials and Methods

**Figure 1:**
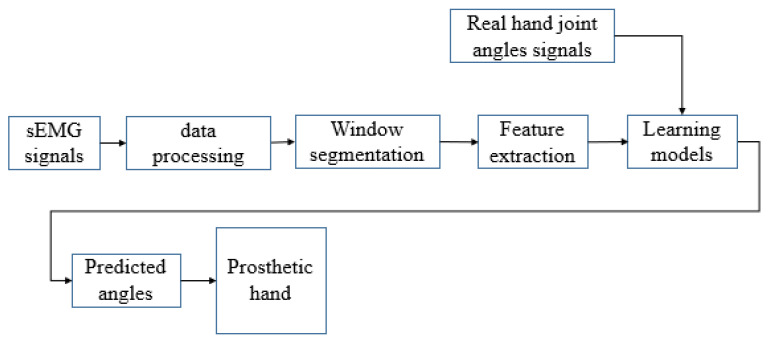
The proposed control scheme’s experimental protocol.

The whole angle decoding system for simultaneous and proportional control is illustrated in Figure 1. The proposed control scheme is combined with three different ensemble learning methods. We use permutation and the combination method to build seven different models to realize accurate angle decoding from sEMG. In order to obtain the label data (the hand joint’s real-time angle) and the related synchronous sEMG data, the research proposes a special experiment that includes three different stages of data acquisition. During the training procedure, different combinations of the three ensemble learning algorithms were tried. In this way, the best model for the real-time angle decoding tasks were chosen. Furthermore, all the experiment data were divided into ten even parts. For testing the performance of the proposed models, one part was used for testing, and the remaining nine parts were used to train the model. This method is also known as 10-fold cross-validation. The resulting model can be used as a drive signal for the myoelectric hand by continuously decoding the finger joint angle from the input forearm sEMG signal.

**Figure 2:**
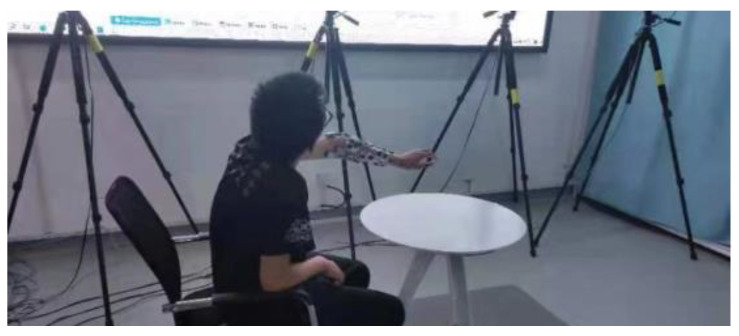
Experiment scene.

**Figure 3:**
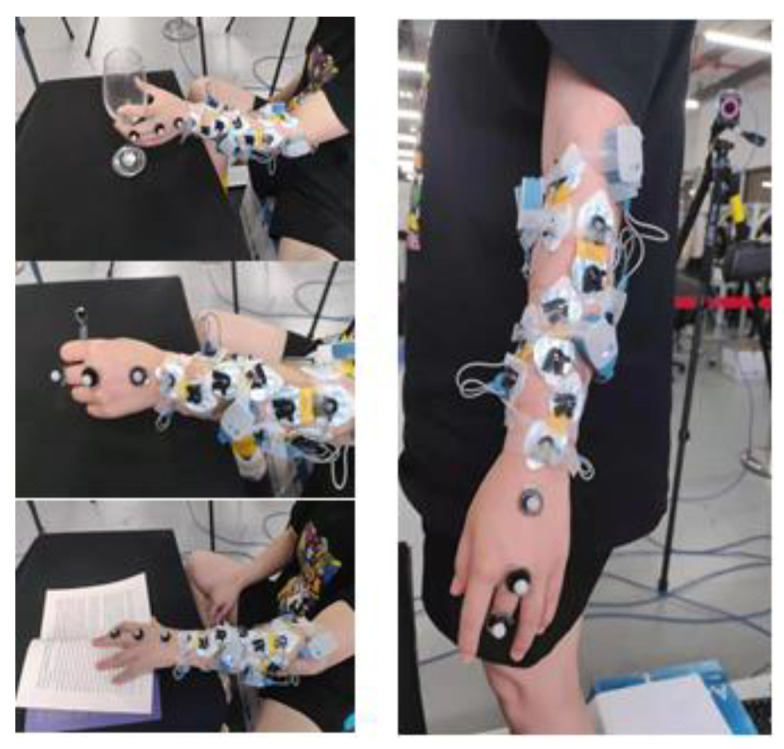
sEMG signal electrode sticking position and grip items are shown.

Ten subjects (age range of 20–30 years old, all right-handed, reference S1-S10) without any musculoskeletal disorders or nervous system diseases participated in our designed experiments. A scene of the experiment is shown in Figure 2. In addition, they had no experience of using the sEMG control technology before the experiment. The position of the sEMG signal electrode adhesion and the grip items are shown in Figure 3.

The study was conducted in accordance with the protocols approved by the research ethics board of Xinjiang University. Before the experiment, we obtained written informed consent from all the subjects and permission to record and publish the photographs for research.

The experiments included simultaneous data acquisition of hand angle data and sEMG data from the subject’s forearm. Eight muscles of the forearm were chosen to collect sEMG signals through the UltiumTM wireless sEMG acquisition system (Noraxon Incorporated, Scottsdale, AZ, USA). The eight muscles studied were abductor pollicis longus, flexor pollicis longus, flexor carpi radialis, flexor digitorum superficialis, flexor digitorum profundus, extensor digitorum, extensor digitorum, and extensor carpi radialis. In our experiments, the corresponding muscles were found through palpation and referring to an anatomical atlas. After decontamination with alcohol, surface electrodes were glued to the muscle belly of the corresponding muscle.

**Figure 4:**
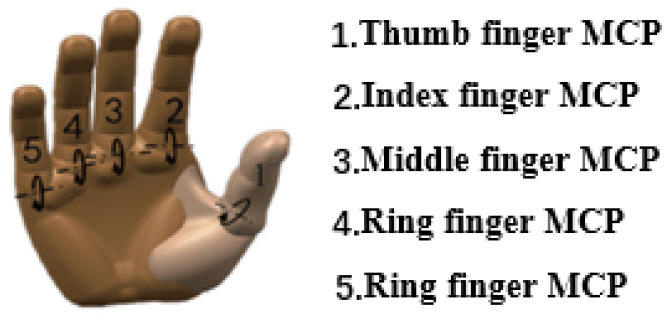
Schematic diagram of the angle of the metacarpophalangeal joint of the finger to be decoded.

For hand joint angles, a Vicon motion system (UK) was used to obtain the related angle data. In our research, metacarpophalangeal (MCP) joint angles were chosen as the estimated target of our model. To undertake this, reflective dots were attached to the metacarpophalangeal joint and two other reflective dots to the ends of the two knuckles that make up the joint. The purpose of this was to create an angle that could be measured. As a result, we could obtain five MCP angles overall. It is very easy to determine that the angular movement of the metacarpophalangeal joint ranges between 90 and 180 degrees. The angle of the metacarpophalangeal joint can be found by applying the inverse cosine formula. The MCP angles’ positions are shown in Figure 4.

Throughout the data collection phase, the subjects were arranged to sit in a comfortable chair with the forearm area supported by a foam box. The purpose of this was to prevent the subject from developing muscle fatigue in the arm, which could affect the purity of the data throughout the experiment. The whole experiment was divided into two phases. The first phase was the independent movement of five fingers. Each finger was moved as substantially as possible in the range of motion for five consecutive minutes. To prevent muscle fatigue, a rest period of approximately five min was set between finger movements. The second phase was finger angle change in the real grasp tasks (see Table 1). Specific grasping items were keyboards, goblets, and books. Because the operation of the human hand on these objects included tapping, grasping and scrolling, which are common hand movements in life. Each time the corresponding object was manipulated or gripped, all the right hand’s fingers were involved in the exercise. Each exercise lasted five min, with a five-minute break in between. Moreover, hand grips were used in specific experiments to carry out the acquisition of sEMG signal values for the eight muscles corresponding to the maximum voluntary contraction force in order to apply normalization for the later data processing. When recording the maximum voluntary contraction force, the subject was asked to exert the maximum force possible and hold it for three seconds. 

During the entire experiment, the optical capture data and the sEMG acquisition data were acquired simultaneously through the hardware system that was equipped in the VICON system.

### 3.1. Data Processing and Feature Extraction

**Figure 5:**
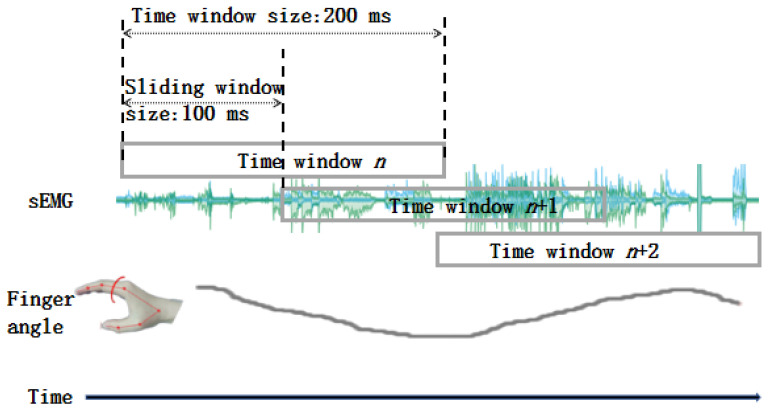
Schematic diagram of the sliding time window.

The sEMG signals underwent a pre-processing stage, including band-pass filtering to eliminate unwanted DC noise, high-frequency noise, and motion artifacts. The filtering was performed off-line using a sixth-order zero-lag Butterworth filter with a band-pass frequency of 15–450 Hz. Additionally, the sEMG signal was comb-filtered at 50 Hz and its integer multiplied to suppress power line interference. In the feature extraction step, eight sEMG features were selected to process the raw sEMG signals; the detailed formulas of these features are shown below. The reason why we chose these eight features is that the time-domain features are very efficient and have low computing requirements. Additionally, the traditional sEMG feature set, TDAR, which consists of Hudgins’ time-domain features [26]. The sEMG signals were divided into overlapping windows of 200 ms with a 50% overlap, which is shown in Figure 5. The reason why we set the sliding window size at 200ms and the overlapping ratio at 50% is that we had investigated the different parameters’ performances in the conference paper [27] and had found this kind of combination to be the best one.

For the finger angle signals, the mean value was extracted from each corresponding processing window. In this research, we chose eight time-domain features to realize finger angles decoding tasks because time-domain features require lower computational resource than frequency-domain features. The formulas used to calculate the features are detailed below.

(1) Mean absolute value (*T*_MAV_) as follows:

(1) $$T_{\mathrm{MAV}}=\frac{1}{N}\sum_{i=1}^{N}\left|x_{i}\right|$$

where *N* refers to the number of sampling points, *x*_i_ represents the sEMG signal value. This feature represents the strength of muscle movement.

(2) Zero-crossing value (*T*_ZC_) as follows:

(2) $$T_{\mathrm{ZC}}=\sum_{i=1}^{N-1}\mathrm{sgn}(-x_{i}x_{i+1})$$

where sgn stands for the symbolic function. This feature can obtain the frequency information from the time domain.

(3) Integral EMG value (*T*_i_) as follows:

(3) $$T_{i}=\sum_{i=1}^{N}|x_{i}|$$

This feature is normally used as an onset detection index in EMG non-pattern recognition and in clinical applications (e.g. Huang & Chen, 1999; Merletti, 1996).

(4) Root mean square (*T*_RMS_) as follows:

(4) $$T_{\mathrm{RMS}}=\sqrt{\frac{1}{N}\sum_{i=1}^{N}x_{i}^{2}}$$

This is modeled as an amplitude-modulated Gaussian random process that is related to the constant force and the non-fatiguing contraction.

(5) Waveform length (*T*_WL_) as follows:

(5) $$T_{\mathrm{WL}}=\sum_{i=1}^{N-1}\left|x_{i+1}-x_{i}\right|$$

This is defined as the cumulative length of the EMG waveform over the time segment.

(6) Log the value of the EMG signal (*T*_LOG_) as follows:

(6) $$T_{\mathrm{LOG}}=\exp[\frac{1}{N}\sum_{i=1}^{N}\log(|x_{i}|)]$$

(7) Positive and negative changes in EMG slope (*T*_SSC_) as follows:

(7) $$T_{\mathrm{SSC}}=\sum_{i=2}^{N-1}f[(x_{i}-x_{i-1})\times (x_{i}-x_{i+1})]$$

(8) $$f(x)=\{\begin{array}{cc} 1,if & x\geq V_{\mathrm{th}}\\ 0, & x<V_{\mathrm{th}} \end{array}$$

where *V*_th_ is the threshold value. This feature is another method used to represent frequency information about the EMG signal.

(8) Willison amplitude (*T*_wamp_) as follows:

(9) $$\begin{array}{l} T_{\mathrm{wamp}}\sum_{i=1}^{N-1}[f(|x_{n}-x_{n-1}|)];\\ f(x)=\{\begin{array}{cc} 1,if & x\geq V_{\mathrm{th}}\\ 0, & otherwise \end{array} \end{array}$$

This is also a measure of the frequency information of the EMG signal.

**Figure 6:**
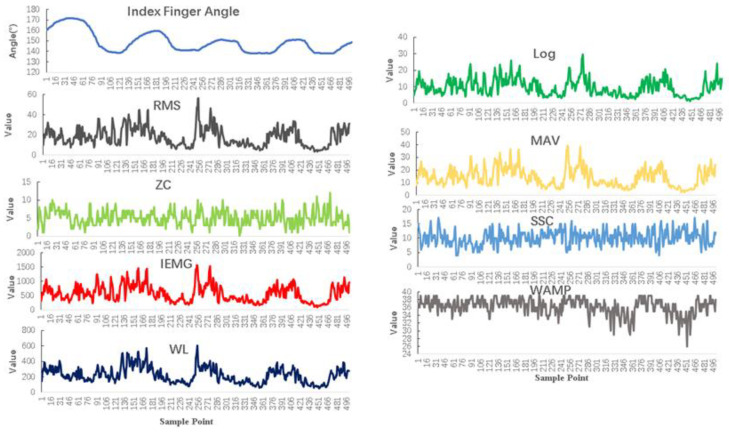
Angle change with change in features.

For better visualization, we have displayed the change in one channel’s features with the index finger’s angle change in Figure 6.

**Figure 7:**
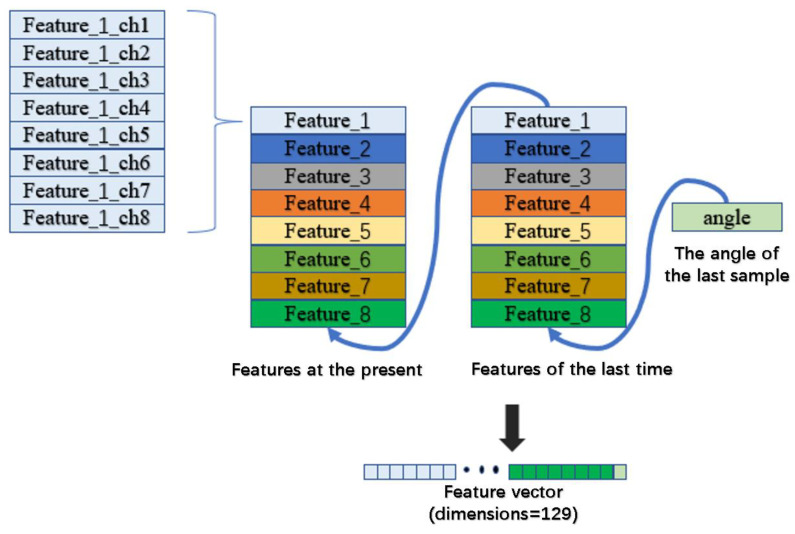
The structure of the feature vector.

The features used in this study were formed by using eight features calculated separately for each channel after stacking the features at this moment with the features at the previous moment and decoding the angle values at the previous moment through the structure shown in Figure 7.

### 3.2. Learning Models

#### 3.2.1. Three Ensemble Learning Methods

For the model selection, we decided to use the ensemble learning method to realize the angle decoding from the sEMG signals. Additionally, the most famous ensemble learning methods in the world may be XGBoost, LightGBM, and CatBoost. Therefore, we decided to use these three methods and their corresponding combinations to build the decoding model. All three of the integrated learning models used are machine learning models based on a gradient boosting tree structure, but the principles used in constructing the leaf nodes are not the same, thereby resulting in different model structures for solving the same problem. The main reasons for the success of deep learning, according to the analysis, are the ability to process features layer by layer at a certain depth and the sufficient structural complexity. The modelling idea employed in this study is that of fusing different integrated learning models through stacking methods so that the deep learning idea can be used to achieve better decoding results.

XGBoost is a gradient boosting algorithm for machine learning [28]. It stands for “eXtreme Gradient Boosting” and is an optimized version of gradient boosting. The trees in XGBoost grow in a level-wise manner. XGBoost undertakes the splits with the specified max_depth hyperparameter and then starts pruning the tree backwards, removing the splits that have no positive gain.

LightGBM is a gradient boosting framework that uses tree-based learning algorithms [29]. It was developed by Microsoft to address the limitations of traditional gradient boosting algorithms, such as slow training speed and poor scalability. LightGBM utilizes a novel tree-growing algorithm, which is called gradient-based one-side sampling (GOSS), to significantly reduce training time and improve model accuracy. Additionally, LightGBM uses a histogram-based representation of data, which speeds up the tree-building process and reduces the memory usage compared with other gradient boosting algorithms. LightGBM selects the leaves with the least growth loss, allowing for trees with asymmetric growth. Because it does not grow level by level but leaf by leaf, overfitting can occur when the data are small. In this case, it is important to control the depth of the model.

CatBoost is a gradient boosting library developed by Yandex, which specializes in processing datasets with a large number of categorical features [30]. It has become popular in recent years due to its efficiency in handling categorical variables, its ease of use, and its high accuracy in real-world applications. The data structure used by CatBoost is a symmetric binary tree. At each level of such a tree, the feature-splitting pair that brings the lowest loss (according to the penalty function) is selected and used for all nodes at that level.

**Figure 8:**
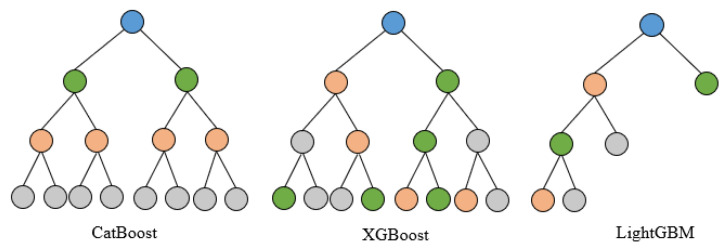
The structure of CatBoost, XGBoost, and LightGBM.

The structures of these three models are illustrated in the Figure 8. The objective function of these three models includes a loss function and a regularization term. The objective function expression starts as follows:

(10) $$Obj(\theta )=\sum_{i=1}^{n}l\left({\hat{y}}_{i},y_{i}\right)+\sum \Omega \left(f_{k}\right)$$

where *l*(*ŷ*_i_,*y*_i_) is used to represent the loss error, *Ω* (*f_k_*) denotes the regularization term to control the complexity of the tree model and prevent overfitting. Three ensemble learning models are all developed based on the gradient boosting tree, so, in actual operation, the residual fitting of each layer of the tree model is derived from the negative gradient of the loss function in the current model. 

All three algorithms above are ensemble learning methods developed based on the boosting method. Using a grid search algorithm, the parameter combination with the smallest mean square error between the predicted values and true values of the test portion was chosen as the optimal one. Their hyperparameters are shown in Table 2.

#### 3.2.2. Stacking Method

**Figure 9:**
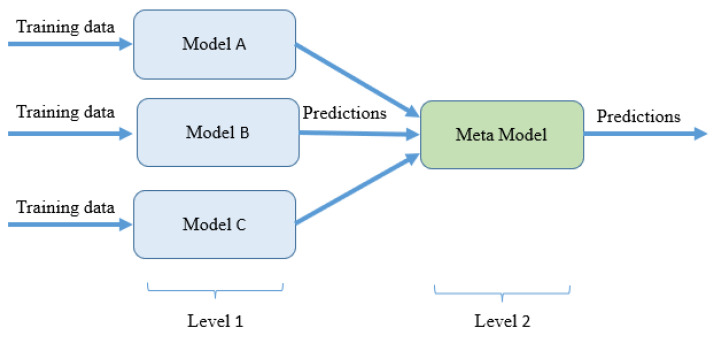
The structure of stacking method.

Stacking is one of the most used and best-performing ensemble techniques used in the field of machine learning. It is very similar to the voting ensembles, but it also assigns the weights to the machine learning algorithms where two layers of models are showed as follows: ground models and meta models. Due to this, stacking tends to perform the better than all the other ensemble techniques used in machine learning. A diagram of the stacking method is shown in Figure 9.

**Figure 10:**
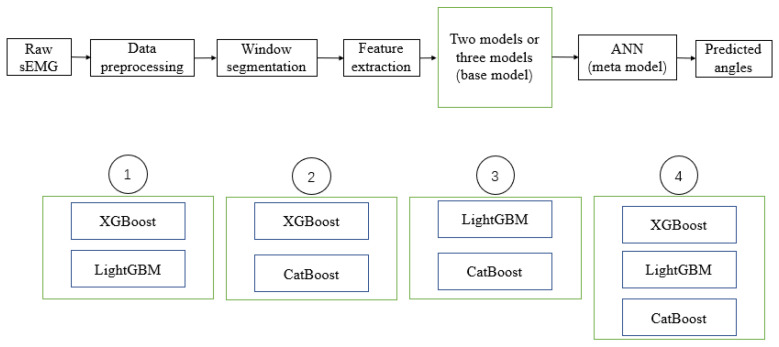
Training and testing strategy of the pipeline of the stacking used for ensemble learning.

In this study, these three methods were first applied to decode the experimental data to check their performance. In order to further improve the performance of the decoded models, the stacking method was thought to be used to combine the three models to form a new integrated regression model, and it was found through experiments that the results were indeed greatly improved. The overall framework diagram for applying the stacking method to these three integrated learning models is shown in Figure 10, with a total of four combinations being used to generate new regression models, where the meta-learner was chosen as a single layer artificial neural network. Additionally, in order to compare the performance of the proposed method and the model that is widely used for the related task, we chose the Gaussian Process as the control group.

A total of 10 subjects were divided into one group containing 5 healthy men aged 22–30 and another containing 5 healthy women aged 23–26, and the subjects were right-handed. Each subject carried out five sessions for one experiment motion. Each session’s data for each subject were divided into ten parts. Nine of these parts were used to train the model, while one of these parts was used to test it. A 10-fold cross-validation method was used to test the proposed model’s performance. For a better fit and to prevent the training from diverging, the training data were standardized to the figure of 0,1.

### 3.3. Performance Index

For the angle regression task, the root mean squared error (RMSE) and Pearson correlation coefficient (CC) were chosen to judge our models’ performances. RMSE is a kind of index which that evaluate the absolute error of the model, while CC is a kind of index that can evaluate the similarity between the model’s prediction and the real results. This is demonstrated as follows:

(11) $$\rho_{X,Y}=\frac{\mathrm{cov}(X,Y)}{\sigma_{X}\sigma_{Y}}=\frac{E((X-\mu_{X})(Y-\mu_{Y})}{\sigma_{X}\sigma_{Y}}=\frac{E(XY)-E(X)E(Y)}{\sqrt{E(X^{2})-E^{2}(X)}\sqrt{E(Y^{2})-E^{2}(Y)}}$$

(12) $$\mathrm{RMSE}=\sqrt{\frac{1}{m}\sum_{i=1}^{m}{\left(y_{i}-{\hat{y}}_{i}\right)}^{2}}$$

where *X* and *Y*, respectively, represent two groups of samples; cov stands for covariance; *E* represents expectation; *σ* represents variance; and *y* and *ŷ*_i_, respectively, represent the real angles and the estimated angles. When the value of *ρ* is greater than 0.7, the model is considered to have good regression performance. When *ρ* = 1, it means that the model output results are completely linear with the actual results. The reason for using CC is to judge the changing trend of the real and the estimated curves.

## 4. Results

In this section, the research compares the estimation performance of the seven models we proposed. We also investigated the different models’ performances on different fingers. Additionally, we tested the individual applicability of different models. In this research, we treated the angle between the MCP bone and the proximal phalanx as the control angle. One sample point of the curve represents 10 milliseconds.

### 4.1. Model Comparison

**Figure 11:**
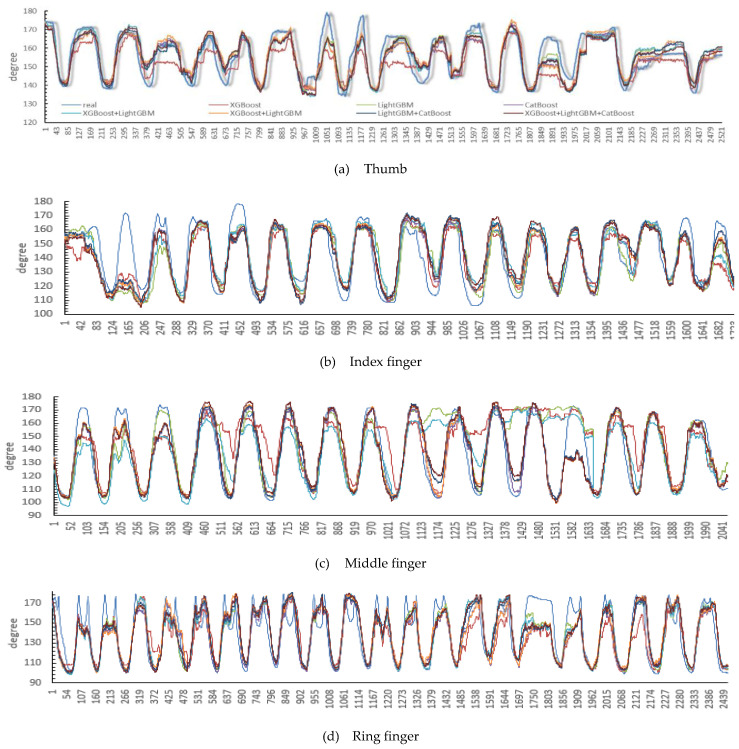

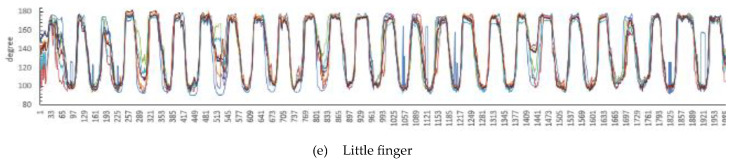
Performance of the different models in finger metacarpophalangeal joint angle estimation (the x-axis represents the sample points).

Seven models were established for all subjects. In this section, their different performances are shown on the same dataset. The overall estimation performance of hand angles using the seven models is shown in Figure 11. For each finger, the average CC values are reported for all 10 participants. The performance index for each participant was averaged over all four stages.

**Figure 12:**
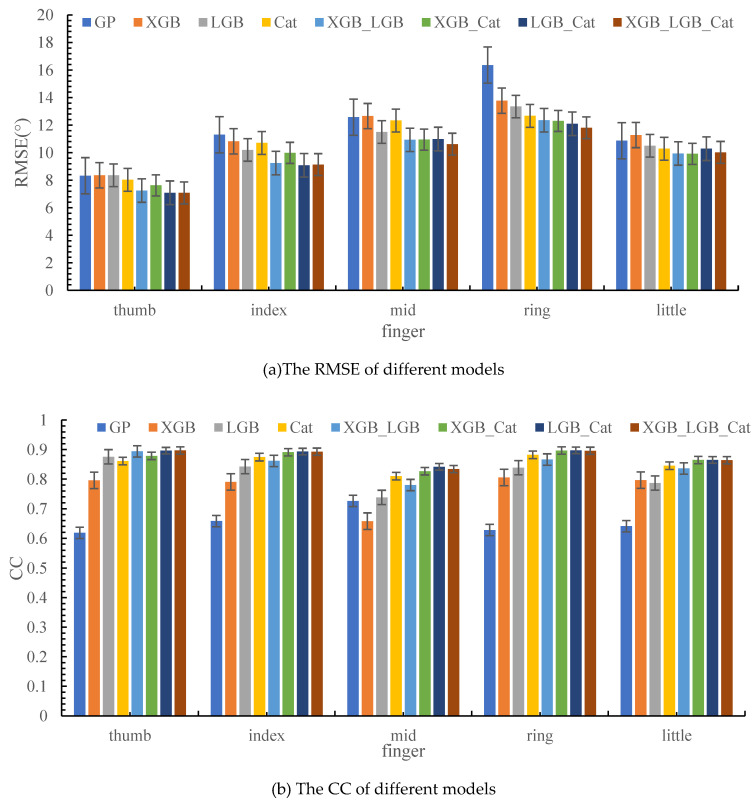
The decoding effect of different finger metacarpophalangeal joint angles.

The estimation performances of all the models are shown in Figure 12 and Table 3, and the model that combines LightGBM with CatBoost achieved the highest accuracy with a CC value of 0.897 ± 0.022. It seems that combination of more accurate models could increase the estimation accuracy, which is confirmed by the fact that the combination of LightGBM and CatBoost achieved higher accuracy. This shows that the stacking method is indeed effective for improving the performance of the model.

### 4.2. Comparison of DOFs

From Figure 11, the middle finger’s performance is the worst in terms of CC. The possible reason for this is that there is a high degree of overlap between the muscles controlling the movement of the middle finger and those controlling the other fingers, thereby resulting in poor recognition accuracy, but a CC value of 0.841 can also be achieved using the stacking model of LightGBM + CatBoost. The average estimation performances using seven models are 0.871 ± 0.033, 0.864 ± 0.035, 0.784 ± 0.061, 0.869 ± 0.032, and 0.837 ± 0.030 for the thumb, index finger, middle finger, ring finger, and little finger, respectively.

### 4.3. Comparison of Different Subjects

**Figure 13:**
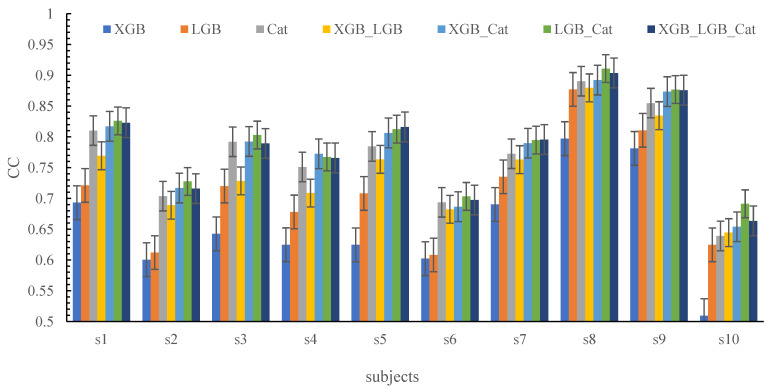
The CC of different subjects’ joint angle estimates against the measurements.

In this research, 10 volunteers were recruited to take part in our experiments. To analyze the generality of our proposed methods, we contrasted the models’ performances on different subjects. The results are shown in Figure 13 and Table 4. The highest accuracy was (CC = 0.911 ± 0.87%) for subject eight through the stacking method that combined LightGBM with CatBoost. The lowest accuracy was (CC = 0.509 ± 0.87%) through XGBoost for subject 10.

## 5. Discussion

In this section, we contrast the three ensemble learning algorithms’ performances in this decoding task. It can be seen that the CatBoost algorithm gives the best results when we do not use the stacking method in building the model. This result shows that the CatBoost model has the best regression function among these three models. LightGBM obtains the second-best result and XGBoost is in third place. By checking the references, we can find that CatBoost uses the symmetric binary tree to build its layers, while LightGBM selects leaves with minimal growth loss, allowing for asymmetrical tree growth; XGBoost’s overall growth strategy is layer growth. Additionally, we think the sEMG signals are more fitting with a symmetric tree structure. For comparing the processing times of different models, we calculate the four basic models’ onefold training times for 20s od experiment data, which are 0.44 s, 0.33 s, 0.40 s and 0.45 s for XGBoost, LightGBM, CatBoost, and GP. When it comes to the stacking models, there are increments of about 0.001s. When running, the processing time for every angle control signal is about 0.001s for XGBoost, LightGBM, and CatBoost, and 0.008 s for GP. Hence, this proposed model is suitable for online control in the future.

In this section, we combine any two ensemble learning models to generate a new integral model. Additionally, we contrast their performance through the same test dataset. We can find the best performance is obtained through the stacking model that is combined LightGBM and CatBoost. This best model’s average CC value is 0.897. More importantly, the order of combining models is not influential because the proposed model has a parallel computational process in the combined part. 

We use all three ensemble models to make a completely new comprehensive model for this task. At first, we imagine that the comprehensive model, which is made of three ensemble learning methods, might be the best one. Yet, the results show us this is not true. Its CC value is 0.897 with the lower RMSE of 7.08. In fact, its performance is very similar to the stacking model combined LightGBM and CatBoost.

As shown in this paper, we can improve the decoding model’s performance by stacking more different ensemble models. If we only implement one ensemble model to realize the decoding task, it may be insufficient.

Because a prosthetic hand has very limited computational resources, the ensemble learning model’s more efficient performance than that of deep learning makes it more suitable for an embedded operating system like the one used in a prosthetic hand.

We have proved that the ensemble learning model based on the tree model demonstrates a much better performance in processing tabular data than that of traditional machine learning methods such as the Gaussian process. Therefore, given that it is a very efficient model, we think this model has great potential for the control of prosthetic hands. The reason why we use ensemble learning methods is that clinical applications need us to implement more efficient and light-weight models to meet the requirements of embodied systems. Additionally, we have undertaken ANOVA to analyze whether age will affect the estimation process, but we have not found any discrimination from the effect of age.

In the future, we will include more subjects with different dominant hands to explore the general performance of the proposed model. In addition, we will deploy our proposed model on an embodied system to test its online performance. More importantly, we will find related amputees to verify the whole system’s efficiency. 

## 6. Conclusions

The research proposes a continuous angle decoding model building scheme that estimates hand angles using sEMG signals recorded from the forearm and validates this scheme in dynamic movements data. The comprehensive model consists of either three different ensemble models or two of them. Additionally, this scheme has been compared with only one kind of the three ensemble learning models. Significant improvement in the correlation coefficient has been found accordingly compared with that found using only one kind of the three ensemble learning models. In this research, we find the comprehensive model that combines CatBoost and LightGBM is the best model for this task, whose average cc value is 0.879 ± 0.022. In this study, all eight features were used for model training, and they represent different information from sEMG. However, in the future, the dimension reduction method will be used to make lighter feature vectors to reduce the computational demand of the model. This paper’s search for the best lightweight decoding model provides a reference and a solution for the other research group to quickly find the best model using stacking methods. Additionally, this study provides a potential solution for precise and robust SPC of hand motion recognition tasks such as applications in the meta universe, teleoperation control, and the use of prosthetic hands.

## Figures and Tables

**Table 1:** Summary of experimental stages.

Stage	Movement	Description	Time of Duration
1	Single finger independent movement	Random free motion	5 × 5min
2	Grasping a USB stick	Natural movement in everyday life	5 min
3	Raising and lowering a wine glass	Natural movement in everyday life	5 min
4	Reading books	Natural movement in everyday life	5 min

**Table 2:** Hyperparameters of the three ensemble models.

	XGBoost	LightGBM	CatBoost
Basic hyperparameters	Max_depth: −1 reg_lambda: 1 × 10^−5^ Subsample: 0.8 colsample_bynode: 0.8 N_estimator: 500	Max_depth: −1 Learning_rate: 0.1 Num_leaves: 31 N_estimators: 100	Max_depth: −1 One_hot_max_size: 10 Iterations: 500 L2_leaf_reg: 1 Learning_rate: 0.1

Note that other unlisted hyperparameters were kept at default values.

**Table 3:** The estimation performance of all models (CC and RMSE).

CC(RMSE)	Thumb	Index Finger	Middle Finger	Ring Finger	Little Finger
GP xgboost	0.618 (8.33) 0.795 (8.36)	0.658(11.3) 0.790 (10.8)	0.726 (12.6) 0.658 (12.6)	0.627 (16.4) 0.805 (13.8)	0.641 (10.9) 0.796 (11.3)
lightgbm	0.875 (8.36)	0.842 (10.2)	0.738 (11.5)	0.838 (13.3)	0.787 (10.5)
catboost	0.861 (8.03)	0.874 (10.7)	0.810 (12.3)	0.882 (12.7)	0.845 (10.3)
Xgboost + lightgbm	0.894 (7.25)	0.861 (9.24)	0.780 (10.9)	0.866 (12.4)	0.836 (9.94)
Xgboost + catboost	0.878 (7.63)	0.890 (9.99)	0.826 (10.9)	0.896 (12.3)	0.864 (9.92)
Lightgbm + catboost	**0.897 (7.09)**	**0.893 (9.09)**	**0.841 (11.0)**	**0.897 (12.1)**	**0.865 (10.3)**
Xgboost + lightgbm + catboost	0.897 (7.08)	0.892 (9.14)	0.834 (10.6)	0.895 (11.8)	0.864 (10.0)

**Table 4:** The estimation performance (CC) of all subjects.

CC	s1	s2	s3	s4	s5	s6	s7	s8	s9	s10
xgboost	0.693	0.600	0.642	0.625	0.625	0.602	0.690	0.797	0.781	0.509
lightgbm	0.721	0.612	0.720	0.678	0.708	0.608	0.735	0.877	0.811	0.625
catboost	0.810	0.704	0.792	0.751	0.784	0.694	0.773	0.890	0.855	0.639
Xgboost + lightgbm	0.769	0.689	0.728	0.709	0.763	0.682	0.763	0.880	0.834	0.644
Xgboost + catboost	0.817	0.717	0.792	0.772	0.806	0.686	0.790	0.892	0.873	0.654
Lightgbm + catboost	**0.826**	**0.728**	**0.803**	**0.767**	**0.813**	**0.703**	**0.795**	**0.911**	**0.877**	**0.691**
Xgboost + Lightgbm + catboost	**0.823**	**0.716**	**0.789**	**0.766**	**0.816**	**0.697**	**0.796**	**0.904**	**0.876**	**0.663**

## Data Availability

The data presented in this study are available on request from the corresponding author (Qing Tao).
